# Anti-Cholera Inoculation

**Published:** 1896-06

**Authors:** J. F. Bennie


### ANTI-CHOLERA INOCULATION.

Recently there have been published in Calcutta. two most important reports on Anti-Cholera Inoculation. One of these is by W. M. Haffkine, the originator of the treatment. Mons. Haffkine is a Roman who has for several years been identified with the Pasteur Institute in Paris. Experimentally, in the laboratory, he has been able to engraft on animals the cholera microbe, and from this source obtain the vaccine.

In nature the virus of infectious diseases is very variable, at one time mild, another time virulent epidemics break out, dependent upon the activity of the virus. Haffkine was able to obtain a fairly uniform degree of virulence in his bacteria, so that his experiments on animals were precise. With the vaccine thus obtained it was demonstrated that animals could with certainty be protected against the fatal infectious disease caused by the cholera bacillus. However instructive these laboratory tests might be, proof of the efficacy of the inoculations on man could only be obtained by practicing them on a large scale in a country where cholera is endemic. The British Secretary of State for India and the Indian government gave Haffkine the fullest possible opportunities to test his vaccines. Whole regiments, both white and native, officers and men, were inoculated. Much interesting work was done in the jails.

In the great city of Calcutta cholera is always present, and here some of the best work was done. As would be expected by all who know Dr. Simpson, the energetic health officer of Calcutta, most important aid was given Haffkine by that gentleman. The second of the reports mentioned is by Dr. Simpson and while containing a little less statistical material than Haffkine's its explanations are fuller and altogether more readable.

"For cholera inoculation there are two vaccines, one mild, the other strong. For a complete vaccination it is necessary to inoculate twice; first of all with mild vaccine which produces some pain at the seat of inoculation, discomfort and fever for about one day; a period of five days is allowed to elapse, and a second inoculation is performed with the second or strong vaccine.

"This second inoculation produces a similar form of malaise to that caused by the first. The discomfort on the whole is milder and of shorter duration than that caused by smallpox." It is completely harmless. The full effect of the vaccination is not obtained until five days after the second operation.

Dr. Simpson, in his excellent report, gives an interesting description of the sanitary abominations of Calcutta. The principal nuisance from a sanitary point of view is constituted by the tanks which "became useful, first, as reservoirs of rain water for supplying the neighborhood, or the surrounding cluster of huts, with water for drinking and cooking purposes, and secondly, as a convenient receptacle into which the drain age of the vicinity should flow."

"Their composition has been officially reported as concentrated London sewage. The drainage of latrines often find an easy and convenient outlet into their waters; soiled clothes of the sick and of the healthy are washed therein; men, women and children bathe and perform their ablutions in the pond, while oxen, buffaloes, horses, goats, and other animals are taken to the water's edge, there given a bath. In such water the inhabitants cleanse their domestic utensils and soak, macerate, and wash their rice and dhal, and not infrequently prepare other kinds of foods."

For years localized outbreaks of cholera have been traced to these tanks.

As might be expected, the neighborhood of tanks presented good fields for Haffkine's investigations. Around two tanks cholera broke out amongst a population of 200, of whom 116 were inoculated. Ten cases of cholera occurred, not one of which was amongst the inoculated.

In 21 houses in Calcutta there were 291 inmates; of these 101 were inoculated and 190 not. Among the 101 inoculated there were no cases of cholera, while among the 190 uninoculated the number of attacks was 29 and of deaths 26.

"Opportunities for comparing the liability to cholera of inoculated with uninoculated living under the same conditions in the same houses presented themselves no fewer than 36 times." "In 36 houses the total number of inmates was 521; of this number 181 were inoculated and 340 not inoculated." The figures are as follows:

| Average number present at date of attack. | | Cases. | Percentage. | Deaths. | Percentage. |
|---|---|---|---|---|---|
| During the first 8 days after inoculation. | | | | | |
| Not inoculated, - - - | 75 | 6 | 8.0 | 4 | 5.33 |
| Inoculated, - - - | 52 | 3 | 5.77 | 3 | 5.77 |
| After eight days. | | | | | |
| Not inoculated, - - - | 265 | 39 | 14.72 | 35 | 13.21 |
| Inoculated, - - - | 140 | 1 | 0.71 | 1 | 0.71 |

The single death among the inoculated in the second list was in a child who had not been vaccinated for the second time, *i. e.*, with the stronger virus, and her only inoculation with the weak virus was made 459 days previous to her death.

It is interesting to note, in reading the particulars of outbreaks of cholera and the effect of inoculations, that in a whole houseful of people who have been inoculated, one or two remain uninoculated, and these one or two are often picked off by the disease, the protected majority remaining untouched.

In some tea gardens in Arsam further experiments were made, of which the following table is sufficient explanation.

From February 9th to April 16th, 1895.

| Non-inoculated | | | Inoculated. | | |
|---|---|---|---|---|---|
| Inhabitants. | Cases. | Deaths. | Inhabitants. | Cases. | Deaths. |
| 1756 | 38 | 16 | 984 | 2 | 1 |
| From April 16 to May 28th. | | | | | |
| 1520 | . 9 | 4 | 1952 | 1.1 | 1.2 |

While enough statistics (we have quoted on y a few of those given in the reports) have not yet been gathered to warrant an absolute conclusion, yet surely the promise for the future is bright and full of hope that cholera may be crushed out at its fountainhead.—J. F. BENNIE, M. D., in *Kansas City Med. Index.*

